# MobiDB: intrinsically disordered proteins in 2021

**DOI:** 10.1093/nar/gkaa1058

**Published:** 2020-11-25

**Authors:** Damiano Piovesan, Marco Necci, Nahuel Escobedo, Alexander Miguel Monzon, András Hatos, Ivan Mičetić, Federica Quaglia, Lisanna Paladin, Pathmanaban Ramasamy, Zsuzsanna Dosztányi, Wim F Vranken, Norman E Davey, Gustavo Parisi, Monika Fuxreiter, Silvio C E Tosatto

**Affiliations:** Dept. of Biomedical Sciences, University of Padua, Via Ugo Bassi 58/B, Padua 35121, Italy; Dept. of Biomedical Sciences, University of Padua, Via Ugo Bassi 58/B, Padua 35121, Italy; Dept. of Science and Technology, Universidad Nacional de Quilmes, Buenos Aires, Argentina; Dept. of Biomedical Sciences, University of Padua, Via Ugo Bassi 58/B, Padua 35121, Italy; Dept. of Biomedical Sciences, University of Padua, Via Ugo Bassi 58/B, Padua 35121, Italy; Dept. of Biomedical Sciences, University of Padua, Via Ugo Bassi 58/B, Padua 35121, Italy; Dept. of Biomedical Sciences, University of Padua, Via Ugo Bassi 58/B, Padua 35121, Italy; Dept. of Biomedical Sciences, University of Padua, Via Ugo Bassi 58/B, Padua 35121, Italy; Interuniversity Institute of Bioinformatics in Brussels, ULB/VUB, Triomflaan, BC building, 6th floor, CP 263, 1050 Brussels, Belgium; Structural Biology Brussels, Vrije Universiteit Brussel, Pleinlaan 2, 1050 Brussels, Belgium; Centre for Structural Biology, VIB, Pleinlaan 2, 1050 Brussels, Belgium; VIB-UGent Center for Medical Biotechnology, VIB, Ghent 9000, Belgium; Department of Biomolecular Medicine, Faculty of Health Sciences and Medicine, Ghent University, Ghent 9000, Belgium; Dept. of Biochemistry, ELTE Eötvös Loránd University, Budapest, Hungary; Interuniversity Institute of Bioinformatics in Brussels, ULB/VUB, Triomflaan, BC building, 6th floor, CP 263, 1050 Brussels, Belgium; Structural Biology Brussels, Vrije Universiteit Brussel, Pleinlaan 2, 1050 Brussels, Belgium; Centre for Structural Biology, VIB, Pleinlaan 2, 1050 Brussels, Belgium; Division of Cancer Biology, The Institute of Cancer Research, 237 Fulham Road, London, SW3 6JB, UK; Dept. of Science and Technology, Universidad Nacional de Quilmes, Buenos Aires, Argentina; Dept. of Biomedical Sciences, University of Padua, Via Ugo Bassi 58/B, Padua 35121, Italy; Dept. of Biomedical Sciences, University of Padua, Via Ugo Bassi 58/B, Padua 35121, Italy

## Abstract

The MobiDB database (URL: https://mobidb.org/) provides predictions and annotations for intrinsically disordered proteins. Here, we report recent developments implemented in MobiDB version 4, regarding the database format, with novel types of annotations and an improved update process. The new website includes a re-designed user interface, a more effective search engine and advanced API for programmatic access. The new database schema gives more flexibility for the users, as well as simplifying the maintenance and updates. In addition, the new entry page provides more visualisation tools including customizable feature viewer and graphs of the residue contact maps. MobiDB v4 annotates the binding modes of disordered proteins, whether they undergo disorder-to-order transitions or remain disordered in the bound state. In addition, disordered regions undergoing liquid-liquid phase separation or post-translational modifications are defined. The integrated information is presented in a simplified interface, which enables faster searches and allows large customized datasets to be downloaded in TSV, Fasta or JSON formats. An alternative advanced interface allows users to drill deeper into features of interest. A new statistics page provides information at database and proteome levels. The new MobiDB version presents state-of-the-art knowledge on disordered proteins and improves data accessibility for both computational and experimental users.

## INTRODUCTION

Intrinsically disordered regions (IDRs) of proteins do not adopt a highly populated structure in isolation, but sample a wide range of conformations. IDR-mediated interactions are implicated in a variety of cellular processes from signal transduction and liquid-liquid phase transition (1–5). IDRs are subjected to extensive pre- and post-translational regulation to modulate protein function in response to cellular stimuli (6–9). Many functions of IDRs, such as entropic springs, flexible linkers or spacers are directly associated with their structural attributes (10,11).

Proteins containing intrinsically disordered regions are present in a considerable fraction of the proteome of eukaryotic organisms (e.g. 43.6% in humans based on MobiDB-lite predictions). In the human proteome, for example, IDRs are highly enriched in interaction interfaces and post-translational modification sites, which serve as regulatory switches of a variety of biochemical pathways (12). Short linear motifs in the IDRs of viruses lead to functional promiscuity and enable compact viral proteomes to extensively rewire their host cells (13). Given the lack of a requirement for a stable globular fold, interfaces in IDRs also appear to have higher evolutionary plasticity allowing them to proliferate in a proteome by ex nihilo evolution. As a result, IDR interfaces are possibly more widespread than ordered interfaces (14). Despite their central roles in key cellular regulatory processes, only a small fraction of disordered regions have been experimentally characterised (15,16). While difficulties in protein expression, purification and structural characterisation hamper experimental characterization, assigning functional modules to dynamic conformational ensembles presents a technical problem for a database.

Manually curated databases, such as DisProt (17,18) and IDEAL (19), collect annotations from the literature and standardise that information. These databases assign the position of IDRs in the sequence, and aim to assemble disorder-related functional annotations. Other databases focus on functional features: DIBS (15) and MFIB (20) collect regions undergoing folding upon binding, and ELM (16) annotates linear motifs. IDRs forming fuzzy complexes are collected in the FuzDB database (21), which also aims to establish links between structural properties and function. Proteins reported to undergo liquid-liquid phase separation (LLPS) typically contain IDRs, which are available in PhasePro (22), PhaSepDB (23) and LLPSDB (24). Altogether, these specialized datasets exhibit a limited overlap, which presents a bottleneck for comprehensive characterisation of the IDRs contained therein (25).

Large-scale identification of IDRs often depends on primary deposition databases of structural data, like the PDB (26), which can be used to indirectly derive disorder information from missing and mobile residues. Although annotation based on this information is less reliable compared to curated resources, it provides a larger set of examples (27,28). A complementary source of information is provided by sequence based predictors, which typically exploit amino acid biases in regions of proteins to identify IDRs (29). Deriving information from these methods requires a critical assessment of the different techniques, which has been implemented in independent blind tests and in the Critical Assessment of Intrinsic protein Disorder (CAID). These efforts, in particular CAID, enable integrating large-scale predicted information on disordered regions into other core data resources such as InterPro (30), UniProtKB (31) and PDBe (26) via MobiDB-lite (32).

In contrast to structural data, functional annotations related to disordered regions are underrepresented in public databases and ontologies (33). This presents a bottleneck for large-scale functional assignment of disordered regions. Functional predictions are in essence limited to regions that fold upon binding, e.g. ANCHOR (34), DISOPRED3 (35) and MoRFCHiBi (66). Recently, prediction of regions that remain disordered upon binding (36) and of regions that exhibit fuzzy binding (37) became available. A new generation of disorder function predictors is also being developed (36) based on the Critical Assessment of Function Annotation challenge (CAFA) (37) and DisProt-based Disorder Ontology (17).

The objective of the new MobiDB (version 4) is to provide stable and sustainable access to data on disordered regions and proteins. As an ELIXIR resource, this is in line with the overall ELIXIR mission and complies with the goals described in the ELIXIR IDP community whitepaper (38). Sustainability has been improved primarily by optimizing the database schema, and by improving the update process and simplifying the data representation formats. The new version incorporates additional curated data from specialized databases and presents novel annotation features including the binding modes derived from processing PDB data as well as an extended list of predictors. It is now possible to download search results and entire genomes with the possibility to specify the format and selected features. A novel statistics page allows the users to compare annotation coverage of different types of annotations and compare the annotation content at the proteome level for all reference proteomes.

## PROGRESS AND NEW FEATURES

### Database content

**Figure 1.**
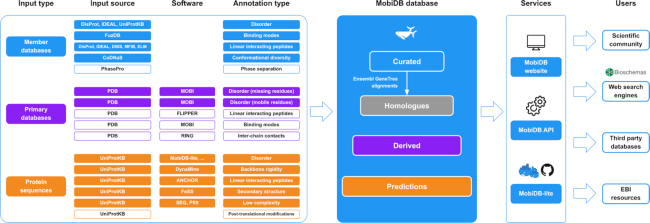
MobiDB data and services. The MobiDB pipeline (left) includes the input source, output features and software used to generate MobiDB data (center). Different background colors indicate different levels of annotation quality. White background in the pipeline (left) indicates novel features, databases and software integrated into MobiDB v4. MobiDB data can be accessed through a website and an API (right). MobiDB predictions can be generated using the MobiDB-lite software which is available both as a Docker container and as a Python package from GitHub. MobiDB web pages are decorated with BioSchemas profiles which allows external search engines to retrieve disorder annotations exploiting structured data.

MobiDB data serves both experimental scientists, who are interested in different aspects of disordered protein regions as well as bioinformaticians, who develop softwares for analysis and prediction of disordered regions. In the new version of the database, several new data resources and features were added to increase the coverage and usability of information about protein disorder. Figure 1 gives an overview of the annotations and services provided by MobiDB. All curated disordered regions are mapped across homologs obtained from GeneTree alignments (39) with >80% of sequence similarity and alignment lengths of 10 residues. This expands the curated set of proteins ten fold. Structural and functional properties of disordered regions are based on third party databases and a set of prediction methods.

The full list of software and resources used in MobiDB is provided in Tables 1 and 2. The different types of annotations and predictions are assembled to provide the user a comprehensive view of properties of disordered regions at the residue level. As compared to the previous releases, MobiDB v4 provides annotations on novel functional aspects of disordered proteins, such as binding modes (36), involvement in post-translational modifications and phase separation (3,4,7).

**Table 1.** Software programs integrated into MobiDB 4. Pfam and Gene3D (*) annotations are retrieved from the InterPro database, all other software are executed locally in the MobiDB server

Method	Input	Features	Reference
Mobi	Structure	Binding modes, missing residues, mobile residues, high temperature residues	(43)
FLIPPER	Structure	Linear interacting peptides	Unpublished
RING	Structure	Residue Interaction Network	(40)
MobiDB-lite	Sequence	Disorder, cysteine rich, proline rich, polar, negative polyelectrolyte, positive polyelectrolyte, polyampholyte	(32)
ESpritz-DisProt	Sequence	Disorder	(54)
ESpritz-NMR	Sequence	Disorder	(54)
ESpritz-Xray	Sequence	Disorder	(54)
IUPred-Long	Sequence	Disorder	(55)
IUPred-Sort	Sequence	Disorder	(55)
VSL2b	Sequence	Disorder	(56)
DisEMBL-465	Sequence	Disorder	(57)
DisEMBL-HotLoops	Sequence	Disorder	(57)
GlobPlot	Sequence	Disorder	(58)
JRONN	Sequence	Disorder	(59)
ANCHOR	Sequence	Linear interacting peptides	(42)
FeSS	Sequence	Secondary structure	(60)
DynaMine	Sequence	Backbone rigidity	(61)
Pfilt	Sequence	Low complexity	(62)
SEG	Sequence	Low complexity	(63)
Gene3D (*)	Sequence	Conserved domains	(64)
Pfam (*)	Sequence	Conserved domains	(65)

**Table 2.** Third party databases integrated into MobiDB 4

Database	Features	URL
CoDNaS	Conformational diversity	http://ufq.unq.edu.ar/codnas/
DIBS	Linear interacting peptides	http://dibs.enzim.ttk.mta.hu/
DisProt	Disorder, linear interacting peptides	https://www.disprot.org/
ELM	Linear interacting peptides	http://elm.eu.org/
FuzDB	Binding modes	http://protdyn-database.org/
IDEAL	Disorder, linear interacting peptides	https://www.ideal-db.org/
MFIB	Linear interacting peptides	http://mfib.enzim.ttk.mta.hu/
PDBe	Protein structures	https://www.ebi.ac.uk/pdbe/
PhasePro	Phase separation	https://phasepro.elte.hu/
UniProtKB	Disorder, transmembrane, coiled coil, signal peptide	https://www.uniprot.org/

Disorder predictions are provided via the MobiDB-lite software (32) over the entire UniProtKB set of protein sequences. In addition, MobiDB calculates annotations from PDB structures, based on manually curated annotations from specific third party databases and propagating manual curation by homology. MobiDB-lite is also integrated for example into InterProScan (30) which propagates its predictions onto several other EBI resources like UniProtKB, InterPro and PDBe.

### Feature classification in MobiDB v4

Different levels of reliability and different features are reported as different and independent annotations. Each MobiDB annotation is identified by an ‘evidence-feature-source’ triplet which uniquely identifies one level of quality, type and source of annotation. Possible values for ‘evidence’ are: (i) ‘curated’, the annotation comes from a manually curated database; (ii) ‘derived’, the annotation is inferred automatically processing primary data, for example PDB structures are processed to detect missing, mobile and lip residues, (iii) ‘homology’, annotations are obtained aligning curated data and (iv) ‘prediction’, annotations are automatically extracted from the protein sequence. The ‘feature’ element of the triplet represents the type (or flavour) of the annotation, for example ‘disorder’, ‘missing_residues’, ‘binding_mode’, ‘lip’, etc. The ‘source’ element represents the annotation source which can be the name of a software, a third party database or the type of consensus. Possible values are: ‘mobidb_lite’, ‘disprot’, ‘merged’, ‘th_90’, etc. The full list and explanation of all available triplets is provided on the about page of the website.

### Novel functional annotations in MobiDB v4

The main difference in MobiDB v4 as compared to the previous releases of MobiDB is the addition of novel functional aspects related to disordered regions, which are listed below and with a white background on the first block of Figure 1. These contribute to establishing links between conformational ensembles and their biological roles.

### Binding modes

Binding modes of disordered regions refer to the conformational transitions of IDRs upon interacting with specific partners. IDRs can undergo disorder-to-order transitions and fold upon the template, or remain disordered (disorder-to-disorder transition) in a partner-bound form (36). Some IDRs exhibit both behaviors, and exhibit context-dependent binding with different partners or cellular conditions (37). These binding modes were assigned from collecting all bound-state experimental evidence from PDB, as defined in (36).

MobiDB implementation identifies inter-chain binding regions and the mono-/multimeric state using the RING software (40). RING generates residue level contact-networks by identifying all types of non-covalent interactions at atomic level in a protein structure (PDB). The residue interaction network generated by RING is combined with data on disorder derived from missing residues. Comparison of disordered residues in free and bound form of the protein is used to define the different binding modes: disorder-to-order, disorder-to-disorder and context-dependent binding.

### Linear interacting peptides (LIPs)

Interactions of disordered regions are realized via short peptide motifs (41). These linear motifs can be collected from the sequence within the Eukaryotic Linear Motif database (16) or predicted using automatic methods such as ANCHOR (42). MobiDB provides a different approach, by exploiting a new software, Fast Linear Interacting Peptides Predictor (FLIPPER), which is able to detect linear interacting peptides (LIPs) from protein structures (PDB). FLIPPER is a random forest classifier which exploits geometrical and physicochemical properties of linear interacting peptides. These properties include linearity, solvent accessibility difference in the bound and unbound state and the ratio of inter- vs. intra-chain contacts. The algorithm has been trained on the same examples used by ANCHOR and validated against DIBS and PixelDB examples. The software is a reimplementation of a published component of the previous MobiDB pipeline (43) and it is freely available from GitHub (https://github.com/BioComputingUP/FLIPPER). After processing the entire PDB, FLIPPER recognized about 6800 different proteins with at least one LIP. Approximately 4.5% of the residues in these proteins are contained in LIPs (see MobiDB statistics) which provide a bona fide dataset for the implementation of novel sequence based predictors and statistical/functional analyses.

### Post-translational modifications

Posttranslational modifications of disordered regions serve as regulatory points of many biochemical pathways (44). Therefore annotating PTM sites is crucial for functional annotation of disordered regions. Data on all types of PTMs are derived from UniProtKB which propagates a small set of well annotated modifications by sequence similarity. Additional phosphorylation sites are provided from the Scop3P database. The Scop3P sites are extracted by large scale re-processing of 36 projects from the PRIDE database (45) with the ionbot approach (https://ionbot.cloud/).

### Liquid-liquid phase separation

Regions forming dynamic liquid droplets are a new facet of disordered proteins (46). Recent data indicates that weak interactions involving disordered regions often govern protein phase separation (47). Although the general biological roles of phase separation remain to be elucidated, systematic annotation of regions undergoing LLPS will contribute to elucidating the underlying sequence-codes.

Regions associated with phase separation processes in MobiDB are derived from the PhasePro database (22). This dataset not only assembles proteins, which were reported to phase separate *in vitro* or *in vivo*, but also specifies those regions, which mediate this process.

### Data generation pipeline and updates

From the technical point of view, the major change is the new format of the entry document in the database. Multi-class annotations, e.g. secondary structure, have been converted to binary classifications by splitting classes into different fields. The grouping of different features on a common field is deprecated since it encodes an arbitrary interpretation of the type of prediction directly in the entry document. Now all predictions appear at the same level and have the same format. It is now possible to integrate new annotations- without refactoring the entry document. This strategy provides a greater flexibility simplifying updates and database maintenance. This enables us to keep MobiDB annotations up to date with UniProtKB releases, with a minimal delay of a few weeks. For new UniProtKB releases MobiDB generates predictions for new sequences by running MobiDB-lite and other tools, updates protein metadata (UniRef IDs, taxonomy, etc.) and removes obsolete entries.

### MobiDB website

The MobiDB website has been completely redesigned in order to improve user experience and satisfy both general use and detailed computational analyses. The graphical user interface retrieves data from a public API which is now well documented and will remain stable for several years.

### Entry page

The entry page has been completely renewed as compared to MobiDB v3 and is now divided in two views: simple and advanced. The simple view of the entry page offers an overview of the entry, focusing on a few annotations. Switching to the advanced view, the user accesses the full collection of data associated with an entry, with dedicated tools for their visualization. In particular, both views revolve around the feature–viewer (48) to organize and dynamically report annotations along the sequence. An interactable network map and a Mol* instance (49) allows an integrated in-depth exploration of residue contacts and structural features.

The feature–viewer combines annotations into a tree of tracks. Parent tracks summarize the information of children tracks by different consensus strategies. Some tracks, e.g. MobiDB-lite tracks are automatically expanded on page load to highlight disordered sub-regions. For each track a squared icon indicates the evidence quality level of the annotation. When a track has children its name gets bold on mouse over and the evidence icon is outlined. On the advanced view, it is possible to customize the feature viewer by hiding specific tracks. By clicking on a region, it is possible to visualize the corresponding annotation in the PDB structure and on the contacts network.

### Statistics page

MobiDB v4 has a new statistics page which provides information about all types of annotations for the entire database and for all proteomes separately. The statistics page provides coverage of a given feature both at the database and proteome level. A search bar on the top allows searching for an organism by providing the identifier of the reference proteome or a string which is evaluated against the organism name and kingdom.

### Searching and downloading data

The browse page is designed upon the MobiDB server API. MobiDB provides programmatic access to perform a search through a RESTful web service API. A single entry can be retrieved by using UniProtKB identifiers, while database searches can be performed by specifying query fields directly as URL parameters in the HTTP request. Protein metadata (UniProtKb accession, gene, organism, NCBI taxon ID, etc.) can be searched for exact matches, whereas the disorder content and sequence length accept a range of values. In the case of a free text search (free_text parameter) the server uses the EBI Proteins API (50) to retrieve proteins containing the input string in the protein name. In that case the result order is provided by the UniProtKB annotation score (https://www.uniprot.org/help/annotation_score). Both from the browse page and from the API it is possible to download the full search result in different formats, TSV, FASTA and JSON. Entire genomes can be searched and downloaded in minutes.

### BioSchemas

To increase MobiDB interoperability and findability, key web pages are decorated with BioSchemas markup (https://bioschemas.org) (51) developed within ELIXIR, the European infrastructure for biological data. BioSchemas extends Schema.org (https://schema.org) for life sciences making the data contained in the database indexable by search engines and other services such as the Google dataset search tool. MobiDB uses DataCatalog and Dataset profiles in the main page, and DataRecord for the entry pages. The DataRecord includes Protein and SequenceAnnotation profiles. These profiles were recently implemented in MobiDB which makes the database, together with DisProt, among the first resources exposing sequence region information using BioSchemas.org.

## CONCLUSIONS AND FUTURE WORK

The rapidly accumulating experimental data on the structure and function of disordered protein regions highlights the importance of systematic analysis of protein functions related to protein disorder. MobiDBv4 provides a major improvement in this respect as compared to previous releases by adding descriptions of functional aspects of disorder, such detailed information about different binding modes, including both disorder-to-order and fuzzy binding; posttranslational modifications; and regions associated with phase separation processes. Interpretation of residue interaction networks is also facilitated by visualising contact maps in an interactive manner. The database data and programming infrastructure has also been upgraded, facilitating systematic analysis, searches and updates. The new website provides more comprehensive information of each entry in a convenient format for both experimental and computational users.

MobiDB aims to provide gold standard disorder predictions, learning from the results of the Critical Assessment of Intrinsic protein Disorder (CAID, http://idpcentral.org/caid) and the Disorder Ontology prediction challenge at the Critical Assessment of protein Function Annotation (CAFA, https://www.biofunctionprediction.org/cafa). These initiatives will further contribute to the improvement of the disorder predictions available in MobiDB. MobiDB predictions are also integrated into several EBI resources including InterPro, UniProtKB and PDBe. MobiDB is a central resource for the IDP community in ELIXIR.

Thanks to the statistics page, for the first time MobiDB allows to answer questions like for example which is the fraction of disordered residues in a given organism, which is the fraction of residues annotated by the majority of predictors or how many proteins have at least one disordered region.

Further developments of the database aim to improve predictions related to protein disorder. On the one hand, the sequence-based predictions of disordered regions have to be standardised and improved, including the predictions of regions, which can undergo liquid–liquid phase separation. These predictions can be facilitated by evolutionary analysis of disordered regions and vice versa prediction of disordered regions can facilitate evolutionary analysis of proteins with low degrees of sequence similarities (52,53).

The other major line of development is functional predictions for disordered regions, including their roles in interaction networks, conformational switches and organisers of liquid droplets. The novel annotations implemented in MobiDB v4 contribute to these efforts. These functional annotations will also be integrated into CAID and CAFA challenges, and will be related to GO classifications. In the present form, MobiDB provides up-to-date knowledge on disordered proteins and regions, which can be widely used by both experimental and computational experts as well as by third party services.

## References

[1] Ivarsson Y., Jemth P. Affinity and specificity of motif-based protein-protein interactions. Curr. Opin. Struct. Biol. 2019; 54:26–33. 30368054 10.1016/j.sbi.2018.09.009

[2] Olsen J.G., Teilum K., Kragelund B.B. Behaviour of intrinsically disordered proteins in protein-protein complexes with an emphasis on fuzziness. Cell. Mol. Life Sci. CMLS. 2017; 74:3175–3183. 28597296 10.1007/s00018-017-2560-7 PMC5533869

[3] Mitrea D.M., Kriwacki R.W. Phase separation in biology; functional organization of a higher order. Cell Commun. Signal. CCS. 2016; 14:1. 26727894 10.1186/s12964-015-0125-7 PMC4700675

[4] Martin E.W., Mittag T. Relationship of sequence and phase separation in protein low-complexity regions. Biochemistry. 2018; 57:2478–2487. 29517898 10.1021/acs.biochem.8b00008 PMC6476794

[5] Wright P.E., Dyson H.J. Intrinsically disordered proteins in cellular signalling and regulation. Nat. Rev. Mol. Cell Biol. 2015; 16:18–29. 25531225 10.1038/nrm3920 PMC4405151

[6] Bah A., Forman-Kay J.D. Modulation of intrinsically disordered protein function by post-translational modifications. J. Biol. Chem. 2016; 291:6696–6705. 26851279 10.1074/jbc.R115.695056 PMC4807257

[7] Csizmok V., Forman-Kay J.D. Complex regulatory mechanisms mediated by the interplay of multiple post-translational modifications. Curr. Opin. Struct. Biol. 2018; 48:58–67. 29100108 10.1016/j.sbi.2017.10.013

[8] Weatheritt R.J., Gibson T.J. Linear motifs: lost in (pre)translation. Trends Biochem. Sci. 2012; 37:333–341. 22705166 10.1016/j.tibs.2012.05.001

[9] Van Roey K., Gibson T.J., Davey N.E. Motif switches: decision-making in cell regulation. Curr. Opin. Struct. Biol. 2012; 22:378–385. 22480932 10.1016/j.sbi.2012.03.004

[10] Tompa P. The interplay between structure and function in intrinsically unstructured proteins. FEBS Lett. 2005; 579:3346–3354. 15943980 10.1016/j.febslet.2005.03.072

[11] van der Lee R., Buljan M., Lang B., Weatheritt R.J., Daughdrill G.W., Dunker A.K., Fuxreiter M., Gough J., Gsponer J., Jones D.T. et al. Classification of intrinsically disordered regions and proteins. Chem. Rev. 2014; 114:6589–6631. 24773235 10.1021/cr400525m PMC4095912

[12] Tompa P., Davey N.E., Gibson T.J., Babu M.M. A million peptide motifs for the molecular biologist. Mol. Cell. 2014; 55:161–169. 25038412 10.1016/j.molcel.2014.05.032

[13] Davey N.E., Travé G., Gibson T.J. How viruses hijack cell regulation. Trends Biochem. Sci. 2011; 36:159–169. 21146412 10.1016/j.tibs.2010.10.002

[14] Davey N.E., Cyert M.S., Moses A.M. Short linear motifs - ex nihilo evolution of protein regulation. Cell Commun. Signal. CCS. 2015; 13:43. 26589632 10.1186/s12964-015-0120-z PMC4654906

[15] Schad E., Fichó E., Pancsa R., Simon I., Dosztányi Z., Mészáros B. DIBS: a repository of disordered binding sites mediating interactions with ordered proteins. Bioinforma. Oxf. Engl. 2018; 34:535–537. 10.1093/bioinformatics/btx640 PMC5860366 29385418

[16] Kumar M., Gouw M., Michael S., Sámano-Sánchez H., Pancsa R., Glavina J., Diakogianni A., Valverde J.A., Bukirova D., Čalyševa J. et al. ELM—the eukaryotic linear motif resource in 2020. NucleicAcidsRes. 2020; 48:D296–D306. 10.1093/nar/gkz1030 PMC7145657 31680160

[17] Hatos A., Hajdu-Soltész B., Monzon A.M., Palopoli N., Álvarez L., Aykac-Fas B., Bassot C., Benítez G.I., Bevilacqua M., Chasapi A. et al. DisProt: intrinsic protein disorder annotation in 2020. NucleicAcidsRes. 2020; 48:D269–D276. 10.1093/nar/gkz975 PMC7145575 31713636

[18] Piovesan D., Tabaro F., Mičetić I., Necci M., Quaglia F., Oldfield C.J., Aspromonte M.C., Davey N.E., Davidović R., Dosztányi Z. et al. DisProt 7.0: a major update of the database of disordered proteins. Nucleic Acids Res. 2017; 45:D1123–D1124. 27965415 10.1093/nar/gkw1279 PMC5210598

[19] Fukuchi S., Amemiya T., Sakamoto S., Nobe Y., Hosoda K., Kado Y., Murakami S.D., Koike R., Hiroaki H., Ota M. IDEAL in 2014 illustrates interaction networks composed of intrinsically disordered proteins and their binding partners. Nucleic Acids Res. 2014; 42:D320–D325. 24178034 10.1093/nar/gkt1010 PMC3965115

[20] Fichó E., Reményi I., Simon I., Mészáros B. MFIB: a repository of protein complexes with mutual folding induced by binding. Bioinforma. Oxf. Engl. 2017; 33:3682–3684. 10.1093/bioinformatics/btx486 PMC5870711 29036655

[21] Miskei M., Antal C., Fuxreiter M. FuzDB: database of fuzzy complexes, a tool to develop stochastic structure-function relationships for protein complexes and higher-order assemblies. Nucleic Acids Res. 2017; 45:D228–D235. 27794553 10.1093/nar/gkw1019 PMC5210525

[22] Mészáros B., Erdős G., Szabó B., Schád É., Tantos Á., Abukhairan R., Horváth T., Murvai N., Kovács O.P., Kovács M. et al. PhaSePro: the database of proteins driving liquid–liquid phase separation. Nucleic Acids Res. 2020; 48:D360–D367. 31612960 10.1093/nar/gkz848 PMC7145634

[23] You K., Huang Q., Yu C., Shen B., Sevilla C., Shi M., Hermjakob H., Chen Y., Li T. PhaSepDB: a database of liquid-liquid phase separation related proteins. Nucleic Acids Res. 2020; 48:D354–D359. 31584089 10.1093/nar/gkz847 PMC6943039

[24] Li Q., Peng X., Li Y., Tang W., Zhu J., Huang J., Qi Y., Zhang Z. LLPSDB: a database of proteins undergoing liquid-liquid phase separation in vitro. Nucleic Acids Res. 2020; 48:D320–D327. 31906602 10.1093/nar/gkz778 PMC6943074

[25] Necci M., Piovesan D., Tosatto S.C.E. Where differences resemble: sequence-feature analysis in curated databases of intrinsically disordered proteins. Database J. Biol. Databases Curation. 2018; 2018:bay127. 10.1093/database/bay127 PMC6301333 30576490

[26] Mir S., Alhroub Y., Anyango S., Armstrong D.R., Berrisford J.M., Clark A.R., Conroy M.J., Dana J.M., Deshpande M., Gupta D. et al. PDBe: towards reusable data delivery infrastructure at protein data bank in Europe. Nucleic. Acids. Res. 2018; 46:D486–D492. 29126160 10.1093/nar/gkx1070 PMC5753225

[27] Monzon A.M., Necci M., Quaglia F., Walsh I., Zanotti G., Piovesan D., Tosatto S.C.E. Experimentally Determined Long Intrinsically Disordered Protein Regions Are Now Abundant in the Protein Data Bank. Int. J. Mol. Sci. 2020; 21:4496. 10.3390/ijms21124496 PMC7349999 32599863

[28] Varadi M., Kosol S., Lebrun P., Valentini E., Blackledge M., Dunker A.K., Felli I.C., Forman-Kay J.D., Kriwacki R.W., Pierattelli R. et al. pE-DB: a database of structural ensembles of intrinsically disordered and of unfolded proteins. Nucleic Acids Res. 2014; 42:D326–D335. 24174539 10.1093/nar/gkt960 PMC3964940

[29] Das R.K., Pappu R.V. Conformations of intrinsically disordered proteins are influenced by linear sequence distributions of oppositely charged residues. Proc. Natl. Acad. Sci. U.S.A. 2013; 110:13392–13397. 23901099 10.1073/pnas.1304749110 PMC3746876

[30] Mitchell A.L., Attwood T.K., Babbitt P.C., Blum M., Bork P., Bridge A., Brown S.D., Chang H.-Y., El-Gebali S., Fraser M.I. et al. InterPro in 2019: improving coverage, classification and access to protein sequence annotations. Nucleic Acids Res. 2019; 47:D351–D360. 30398656 10.1093/nar/gky1100 PMC6323941

[31] UniProt Consortium UniProt: a worldwide hub of protein knowledge. Nucleic Acids Res. 2019; 47:D506–D515. 30395287 10.1093/nar/gky1049 PMC6323992

[32] Necci M., Piovesan D., Dosztányi Z., Tosatto S.C.E. MobiDB-lite: fast and highly specific consensus prediction of intrinsic disorder in proteins. Bioinformatics. 2017; 33:1402–1404. 28453683 10.1093/bioinformatics/btx015

[33] Necci M., Piovesan D., Tosatto S.C.E. Large-scale analysis of intrinsic disorder flavors and associated functions in the protein sequence universe. Protein Sci. Publ. Protein Soc. 2016; 25:2164–2174. 10.1002/pro.3041 PMC5119570 27636733

[34] Mészáros B., Simon I., Dosztányi Z. Prediction of protein binding regions in disordered proteins. PLoS Comput. Biol. 2009; 5:e1000376. 19412530 10.1371/journal.pcbi.1000376 PMC2671142

[35] Jones D.T., Cozzetto D. DISOPRED3: precise disordered region predictions with annotated protein-binding activity. Bioinforma. Oxf. Engl. 2015; 31:857–863. 10.1093/bioinformatics/btu744 PMC4380029 25391399

[36] Miskei M., Horvath A., Vendruscolo M., Fuxreiter M. Sequence-based prediction of fuzzy protein interactions. J. Mol. Biol. 2020; 432:2289–2303. 32112804 10.1016/j.jmb.2020.02.017

[37] Horvath A., Miskei M., Ambrus V., Vendruscolo M., Fuxreiter M. Sequence-based prediction of protein binding mode landscapes. PLoS Comput. Biol. 2020; 16:e1007864. 32453748 10.1371/journal.pcbi.1007864 PMC7304629

[38] Davey N.E., Babu M.M., Blackledge M., Bridge A., Capella-Gutierrez S., Dosztanyi Z., Drysdale R., Edwards R.J., Elofsson A., Felli I.C. et al. An intrinsically disordered proteins community for ELIXIR. F1000Research. 2019; 8:ELIXIR-1753. 10.12688/f1000research.20136.1 PMC6880265 31824649

[39] Vilella A.J., Severin J., Ureta-Vidal A., Heng L., Durbin R., Birney E. EnsemblCompara GeneTrees: Complete, duplication-aware phylogenetic trees in vertebrates. Genome Res. 2009; 19:327–335. 19029536 10.1101/gr.073585.107 PMC2652215

[40] Piovesan D., Minervini G., Tosatto S.C.E. The RING 2.0 web server for high quality residue interaction networks. Nucleic Acids Res. 2016; 44:W367–374. 27198219 10.1093/nar/gkw315 PMC4987896

[41] Pancsa R., Fuxreiter M. Interactions via intrinsically disordered regions: what kind of motifs. IUBMB Life. 2012; 64:513–520. 22535488 10.1002/iub.1034

[42] Dosztányi Z., Mészáros B., Simon I. ANCHOR: web server for predicting protein binding regions in disordered proteins. Bioinformatics. 2009; 25:2745–2746. 19717576 10.1093/bioinformatics/btp518 PMC2759549

[43] Piovesan D., Tosatto S.C.E. Mobi 2.0: an improved method to define intrinsic disorder, mobility and linear binding regions in protein structures. Bioinforma. Oxf. Engl. 2018; 34:122–123. 10.1093/bioinformatics/btx592 28968795

[44] Bah A., Vernon R.M., Siddiqui Z., Krzeminski M., Muhandiram R., Zhao C., Sonenberg N., Kay L.E., Forman-Kay J.D. Folding of an intrinsically disordered protein by phosphorylation as a regulatory switch. Nature. 2015; 519:106–109. 25533957 10.1038/nature13999

[45] Perez-Riverol Y., Csordas A., Bai J., Bernal-Llinares M., Hewapathirana S., Kundu D.J., Inuganti A., Griss J., Mayer G., Eisenacher M. et al. The PRIDE database and related tools and resources in 2019: improving support for quantification data. Nucleic Acids Res. 2019; 47:D442–D450. 30395289 10.1093/nar/gky1106 PMC6323896

[46] Banani S.F., Lee H.O., Hyman A.A., Rosen M.K. Biomolecular condensates: organizers of cellular biochemistry. Nat. Rev. Mol. Cell Biol. 2017; 18:285–298. 28225081 10.1038/nrm.2017.7 PMC7434221

[47] Boeynaems S., Alberti S., Fawzi N.L., Mittag T., Polymenidou M., Rousseau F., Schymkowitz J., Shorter J., Wolozin B., Van Den Bosch L. et al. Protein phase separation: a new phase in cell biology. Trends Cell Biol. 2018; 28:D420–D435. 10.1016/j.tcb.2018.02.004 PMC6034118 29602697

[48] Paladin L., Schaeffer M., Gaudet P., Zahn-Zabal M., Michel P.-A., Piovesan D., Tosatto S.C.E., Bairoch A. The feature viewer: a visualization tool for positional annotations on a sequence. Bioinformatics. 36:3244–3245. 31985787 10.1093/bioinformatics/btaa055

[49] Sehnal D., Rose A.S., Koča J., Burley S.K., Velankar S. Mol*: towards a common library and tools for web molecular graphics. Proceedings of the Workshop on Molecular Graphics and Visual Analysis of Molecular Data, MolVA ’18. 2018; Eurographics Association, Goslar, DEU 29–33.

[50] Nightingale A., Antunes R., Alpi E., Bursteinas B., Gonzales L., Liu W., Luo J., Qi G., Turner E., Martin M. The proteins API: accessing key integrated protein and genome information. Nucleic Acids Res. 2017; 45:W539–W544. 28383659 10.1093/nar/gkx237 PMC5570157

[51] Gray A.J.G., Goble C., Jimenez R.C. From Potato Salad to Protein Annotation. 2017;

[52] Zsolyomi F., Ambrus V., Fuxreiter M. Patterns of dynamics comprise a conserved evolutionary trait. J. Mol. Biol. 2020; 432:497–507. 31783068 10.1016/j.jmb.2019.11.007

[53] Marchetti J., Monzon A.M., Tosatto S.C.E., Parisi G., Fornasari M.S. Ensembles from ordered and disordered proteins reveal similar structural constraints during evolution. J. Mol. Biol. 2019; 431:1298–1307. 30731089 10.1016/j.jmb.2019.01.031

[54] Walsh I., Martin A.J.M., Di Domenico T., Tosatto S.C.E. ESpritz: accurate and fast prediction of protein disorder. Bioinformatics. 2012; 28:503–509. 22190692 10.1093/bioinformatics/btr682

[55] Mészáros B., Erdős G., Dosztányi Z. IUPred2A: context-dependent prediction of protein disorder as a function of redox state and protein binding. Nucleic Acids Res. 2018; 46:W329–W337. 29860432 10.1093/nar/gky384 PMC6030935

[56] Peng K., Radivojac P., Vucetic S., Dunker A.K., Obradovic Z. Length-dependent prediction of protein intrinsic disorder. BMC Bioinformatics. 2006; 7:208. 16618368 10.1186/1471-2105-7-208 PMC1479845

[57] Linding R., Jensen L.J., Diella F., Bork P., Gibson T.J., Russell R.B. Protein disorder prediction: implications for structural proteomics. Structure. 2003; 11:1453–1459. 14604535 10.1016/j.str.2003.10.002

[58] Linding R., Russell R.B., Neduva V., Gibson T.J. GlobPlot: exploring protein sequences for globularity and disorder. Nucleic Acids Res. 2003; 31:3701–3708. 12824398 10.1093/nar/gkg519 PMC169197

[59] Yang Z.R., Thomson R., McNeil P., Esnouf R.M. RONN: the bio-basis function neural network technique applied to the detection of natively disordered regions in proteins. Bioinformatics. 2005; 21:3369–3376. 15947016 10.1093/bioinformatics/bti534

[60] Piovesan D., Walsh I., Minervini G., Tosatto S.C.E. FELLS: fast estimator of latent local structure. Bioinformatics. 2017; 33:1889–1891. 28186245 10.1093/bioinformatics/btx085

[61] Cilia E., Pancsa R., Tompa P., Lenaerts T., Vranken W.F. From protein sequence to dynamics and disorder with DynaMine. Nat. Commun. 2013; 4:2741. 24225580 10.1038/ncomms3741

[62] Jones D.T., Swindells M.B. Getting the most from PSI-BLAST. Trends Biochem. Sci. 2002; 27:161–164. 11893514 10.1016/s0968-0004(01)02039-4

[63] Wootton J.C. Non-globular domains in protein sequences: automated segmentation using complexity measures. Comput. Chem. 1994; 18:269–285. 7952898 10.1016/0097-8485(94)85023-2

[64] Lewis T.E., Sillitoe I., Dawson N., Lam S.D., Clarke T., Lee D., Orengo C., Lees J. Gene3D: extensive prediction of globular domains in proteins. Nucleic Acids Res. 2018; 46:D435–D439. 29112716 10.1093/nar/gkx1069 PMC5753370

[65] El-Gebali S., Mistry J., Bateman A., Eddy S.R., Luciani A., Potter S.C., Qureshi M., Richardson L.J., Salazar G.A., Smart A. et al. The Pfam protein families database in 2019. Nucleic Acids Res. 2019; 47:D427–D432. 30357350 10.1093/nar/gky995 PMC6324024

[66] Malhis N., Wong E.T.C., Nassar R., Gsponer J. Computational identification of MoRFs in protein sequences using hierarchical application of Bayes rule. PLoS One. 2015; 10:e0141603. 26517836 10.1371/journal.pone.0141603 PMC4627796

